# Optical coherence tomography in healthy human subjects in the setting of prolonged dark adaptation

**DOI:** 10.1038/s41598-023-30747-0

**Published:** 2023-03-06

**Authors:** Erin H. Su, Niranjana Kesavamoorthy, Hossein Ameri

**Affiliations:** Department of Ophthalmology, USC Roski Eye Institute, Keck School of Medicine, University of Southern California, Los Angeles, CA 90033 USA

**Keywords:** Medical research, Health care, Disease prevention, Medical imaging

## Abstract

Human studies have established that short periods of dark adaptation can induce outer retinal thinning and various band intensity changes that can be detected with Optical Coherence Tomography (OCT). Similar findings were observed in mice, including a positive correlation between the degree of outer retinal changes and dark adaptation duration. We decided to assess potential retinal structural changes following prolonged dark adaptation in humans. 40 healthy subjects without any ocular diseases participated in this study. For each subject, one eye was covered for dark adaptation for four hours, and the other eye was left uncovered as a control. Before and after the dark adaptation period, both eyes were assessed with OCT. Using the Heidelberg Spectralis system, basic statistical functions, and qualitative and quantitative analysis, we were able to compare retinal layer thicknesses and band intensities between covered (dark adapted) versus uncovered (control) eyes. Prolonged dark adaptation did not induce any significant thickness, volume, or intensity changes in the outer retina or in the inner or overall retina. These observations thus alter our current understanding of the mechanisms underlying dark adaptation’s neuroprotective effects in preventing blindness and require further study.

## Introduction

Dark adaptation describes the recovery of visual sensitivity in the dark after light exposure. Multiple processes occur simultaneously to allow for retinal homeostasis, including modulation of blood flow^1^, interphotoreceptor matrix protein and photoreceptor signaling protein redistribution^2,3^, and metabolic energy flow reversal^4^. Because visual perception in the dark is rod-mediated and affected by rod photoreceptor health and homeostasis, delayed dark adaptation is often an early symptom of various retinal diseases, including retinitis pigmentosa^5^, diabetic retinopathy^6^, and age-related macular degeneration^7^. Deeper understanding of dark adaptation, as well as early clinical detection, are therefore essential to prevent further irreversible vision loss in these subjects^8^.

Previous animal studies have established that dark adaptation induces outer retinal structural changes related to maintaining retinal homeostasis^8–13^. Concordant studies that have focused on human subjects have shown similar results, mostly in diseased retinas (i.e. diabetic retinopathy, macular diseases, etc.); considerably fewer studies, however, have been performed on healthy human or mouse retinas, and those that have used bleaching or flash light stimuli^9,14–20^.

An ideal imaging modality for studying such structural changes is Optical Coherence Tomography (OCT), a noninvasive, high-resolution, in vivo imaging modality used for monitoring and diagnosing retinal diseases. OCT takes images of optical tissue sections using an infrared light source and has been used for time-lapse retinal imaging to study light-induced morphologic changes.

Many studies have shown the utility of OCT for studying retinal structural changes, especially in the context of dark adaptation, though the dark adaptation periods in these and other clinical studies were done over short periods of time only^21^. To the best of our knowledge, the longest studied period of dark adaptation in human subjects has been 30 min^22^. Given previous mouse study findings of more marked structural changes with increased periods of dark adaptation^9^, our study sought to assess retinal structural changes following prolonged dark adaptation in humans and explore its potential clinical significance.

## Results

The participants’ mean age was 27.65 years (Table 1; 21 subjects male, 19 subjects female). Using high-resolution OCT imaging, we examined healthy human retinal structure under dark adaptation and control conditions.

**Table 1:** Subject demographics. Includes age, sex, best corrected visual acuity (BCVA), refraction (sphere) for each eye, and which eye was dark adapted/patched (^a^excluded from analysis).

OCT participant #	Age	Sex	BCVA	Refraction (SPH)	DA/Patched eye
1	25	F	20/20 OU	− 2.75 OD, − 3.5 OS	OS
2	25	F	20/20 OU	0 OU	OD
3	25	F	20/20 OU	− 5 OU	OD
4	25	M	20/20 OU	0 OU	OS
5	26	M	20/20 OU	− 2.5 OU	OD
6	29	M	20/20 OU	− 6.5 OU	OS
7	26	F	20/20 OU	− 5.5 OD, − 4.5 OS	OS
8	30	M	20/20 OU	− 2 OD, 0 OS	OD
9	33	M	20/20 OU	− 4.5 OU	OD
10	25	M	20/20 OU	− 2.5 OU	OS
11	27	M	20/20 OU	0 OU	OS
12	26	M	20/20 OU	− 4.25 OD, − 4.5 OS	OS
13	25	F	20/20 OU	− 2.5 OU	OD
14	29	M	20/20 OU	0 OU	OD
15	25	F	20/20 OU	− 8.25 OU	OS
16	19	F	20/20 OU	− 3.25 OD, − 2.75 OS	OD
17	23	F	20/20 OU	− 1.25 OD, − 2.5 OS	OS
18	26	M	20/20 OU	− 6 OD, − 5.5 OS	OD
19	24	M	20/20 OU	0 OU	OS
20	31	M	20/20 OU	− 5 OD, − 5.5 OS	OD
21	19	M	20/20 OU	− 1.5 OD, − 1.75 OS	OD
22	28	F	20/20 OU	− 3.75 OD, − 3.25 OS	OS
23	26	M	20/20 OU	0 OU	OD
24	22	F	20/20 OU	0 OU	OS
25	25	F	20/20 OU	− 2 OD, − 3 OS	OD
26	31	M	20/20 OU	− 4.5 OU	OS
27	24	F	20/20 OU	− 2.25 OU	OD
28	28	M	20/20 OU	0 OU	OS
29	25	F	20/20 OU	0 OU	OD
30	25	F	20/20 OU	0 OU	OS
31	22	F	20/20 OU	0 OU	OD
32	25	M	20/20 OU	0 OU	OS
33	28	F	20/20 OU	− 2 OD, − 3 OS	OD
34	30	M	20/20 OU	− 1 OD, − 2 OS	OS
35	31	M	20/20 OU	0 OU	OS
36	29	F	20/20 OU	− 5.75 OU	OD
37	56	M	20/20 OU	− 7 OU	OD
38	60	F	20/20 OU	− 2.75 OD, 0 OS	OS
39^a^	25	F	20/20 OU	− 2.25 OD, − 2.5 OS	OD
40^a^	23	F	20/20 OU	− 4 OD, − 2.25 OS	OS

**Figure 1:**
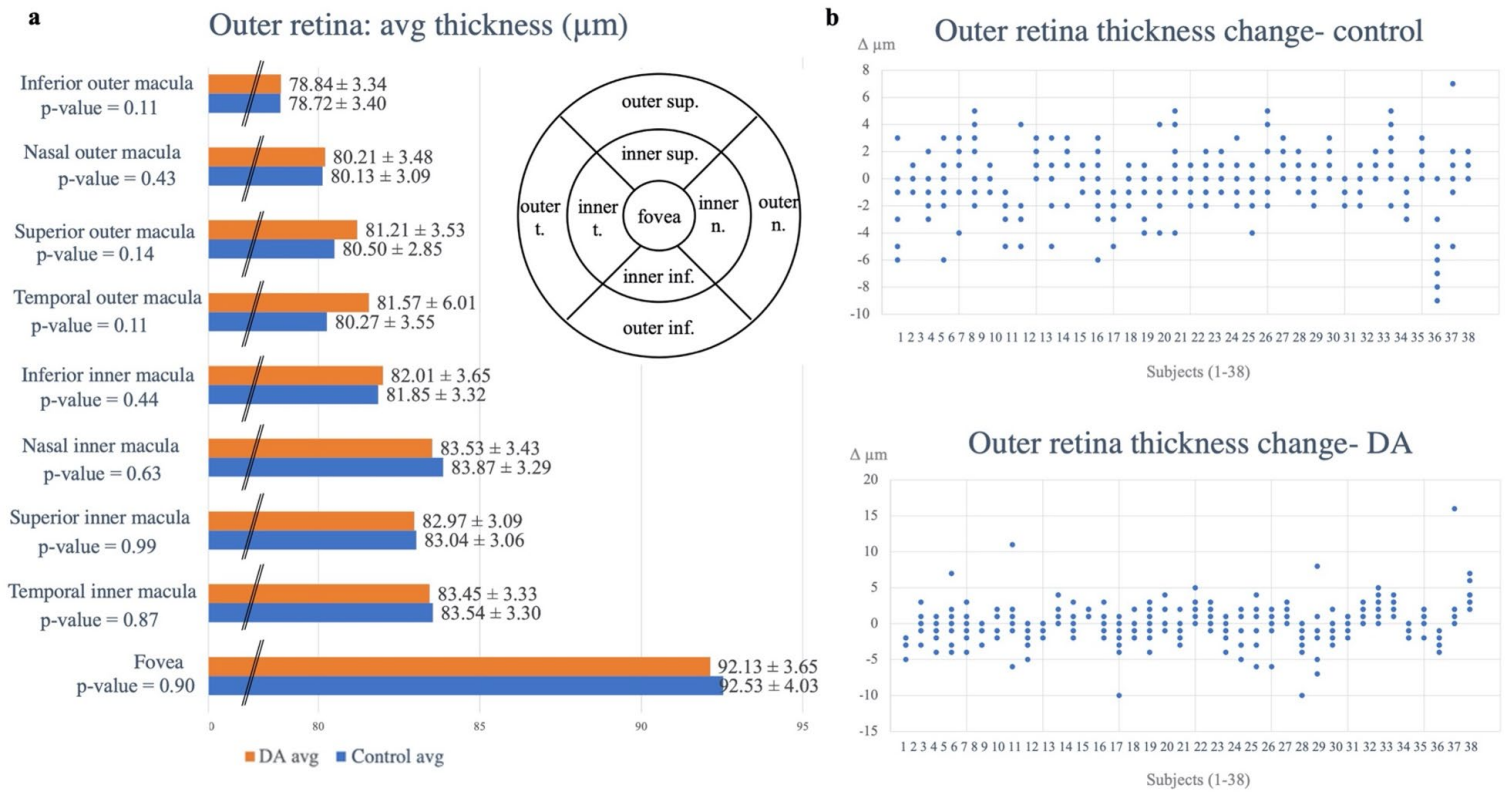
Outer retinal thickness measurements before and after dark adaptation (DA) at baseline and four hours later in the dark adapted and control eyes. (**A**) Bar graph comparing dark adapted (orange) and control (blue) average thickness measurements for the 9 regions of interest (foveal, and inner and outer perifoveal regions) with a labeled thickness map example template included (superior-sup., nasal-n., temporal-t., inferior-inf.). (**B**) Average differences for the outer retinal layer for control and dark adaptation conditions respectively. Data points were taken from the Heidelberg-generated thickness maps from all 9 regions (foveal and inner and outer perifoveal regions) for each subject, and the values before the dark adaptation period were subtracted from those after the dark adaptation period to calculate the difference in values. Negative values show layer thinning and positive values show layer thickening in the setting of dark adaptation. There was no statistically significant difference within the respective nine regions.

**Figure 2:**
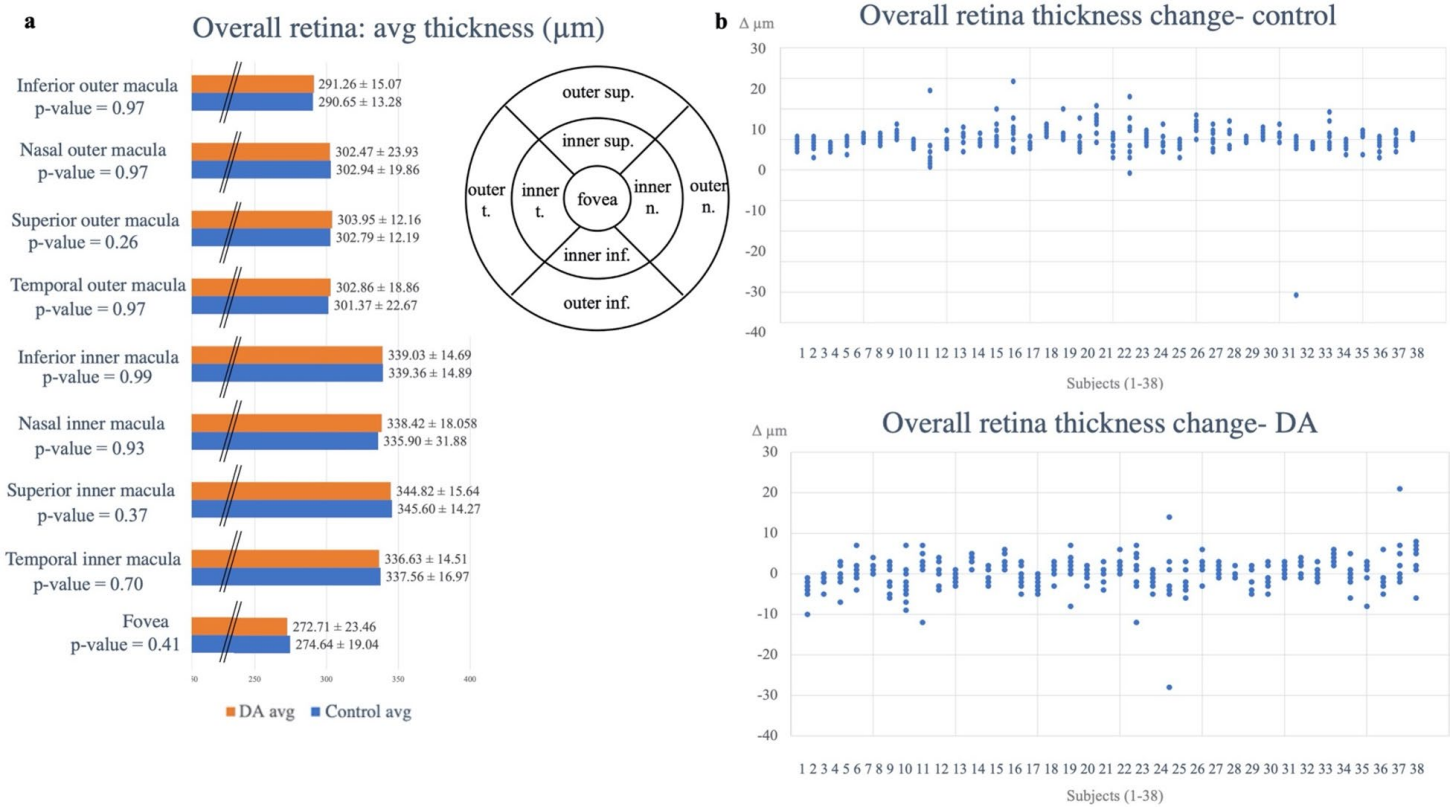
Overall retinal thickness measurements before and after dark adaptation (DA) at baseline and four hours later in the dark adapted and control eyes. (**A**) Bar graph comparing dark adapted (orange) and control (blue) average thickness measurements for the 9 regions of interest (foveal, and inner and outer perifoveal regions), with a labelled thickness map example template included. (**B**) Average differences for the overall retinal layer for control and dark adaptation conditions respectively. Data points were taken from the Heidelberg-generated thickness maps from all 9 regions (foveal and inner and outer perifoveal regions) for each subject, and the values before the dark adaptation period were subtracted from those after the dark adaptation period to calculate the difference in values. Negative values show layer thinning and positive values show layer thickening in the setting of dark adaptation. There was no statistically significant difference within the respective nine regions.

Average thicknesses and volumes of the three retinal layers were calculated across the foveal, and four inner and four outer perifoveal regions (Table 2, Supplemental Table 2). The average thickness values for the outer, inner, and overall retina for dark adaptation and controlled conditions are represented in Fig. 1. Neither thickness nor volume parameters showed any significant changes between any of the three analyzed layers when comparing control versus dark adapted eyes (Figs. 1, 2, Tables 2, 3, Supplemental Figs. 1–4, Supplemental Tables 1, 2); the control and dark adaptation conditions yielded similar results across the overall, inner, and outer retinal layers. Thickness and volume differences across all nine regions (foveal, inner and outer perifoveal) mostly converged upon zero, with both layer thickening and thinning occurring (Figs. 1B, 2B, Supplemental Figs. 1–4B). Paired t-testing using the absolute values of the thickness and volume differences also yielded results that were not statistically significantly different; calculations resulted in p-values of 0.89, 0.35, and 0.20 for overall, inner, and outer retinal layer thickness changes, and p-values of 0.39, 0.14, and 0.33 for overall, inner, and outer retinal layer volume changes respectively (Table 3, Supplemental Table 2).

**Table 2:** Average thickness measurements and p-values for all 9 regions of interest (foveal and inner and outer perifoveal regions) between control and dark adaptation (DA) conditions.

Region of interest	Avg thickness (µm)	Overall retina	Inner retina	Outer retina
Fovea	DA	272.71 ± 23.46	180.74 ± 24.14	92.13 ± 3.65
	Control	274.64 ± 19.04	182.02 ± 19.65	92.53 ± 4.03
	p-value	p = 0.41	p = 0. 57	p = 0.90
Temporal inner macula	DA	336.63 ± 14.51	253.14 ± 12.82	83.45 ± 3.33
	Control	337.56 ± 16.97	254.09 ± 15.44	83.54 ± 3.30
	p-value	p = 0. 70	p = 0.60	p = 0.87
Superior inner macula	DA	344.82 ± 15.64	262.01 ± 14.13	82.97 ± 3.09
	Control	345.60 ± 14.27	262.59 ± 12.96	83.04 ± 3.06
	p-value	p = 0.37	p = 0.37	p = 0. 99
Nasal inner macula	DA	338.42 ± 18.06	255.00 ± 16.22	83.53 ± 3.43
	Control	335.90 ± 31.88	254.68 ± 13.61	83.87 ± 3.29
	p-value	p = 0.93	p = 0.91	p = 0.63
Inferior inner macula	DA	339.03 ± 14.69	257.18 ± 13.62	82.01 ± 3.65
	Control	339.36 ± 14.89	258.06 ± 13.31	81.85 ± 3.32
	p-value	p = 0.99	p = 0.16	0.44
Temporal outer macula	DA	302.86 ± 18.68	222.05 ± 18.56	81.57 ± 6.01
	Control	301.37 ± 22.67	221.07 ± 22.00	80.27 ± 3.55
	p-value	p = 0.97	p = 0.93	p = 0.11
Superior outer macula	DA	303.95 ± 12.16	222.87 ± 10.64	81.21 ± 3.53
	Control	302.79 ± 12.19	222.32 ± 11.16	80.50 ± 2.85
	p-value	p = 0.26	p = 0.95	p = 0.14
Nasal outer macula	DA	302.47 ± 23.93	222.53 ± 23.19	80.21 ± 3.48
	Control	302.94 ± 19.86	222.97 ± 19.06	80.13 ± 3.09
	p-value	p = 0.97	p = 0.98	p = 0.43
Inferior outer macula	DA	291.26 ± 15.07	212.63 ± 13.46	78.84 ± 3.34
	Control	290.65 ± 13.28	211.89 ± 12.47	78.72 ± 3.40
	p-value	p = 0.97	p = 0.93	p = 0.11

**Table 3:** Absolute values of average change in thickness, standard deviations, and p-values for overall, inner, and outer retinal layers between control and dark adapted (DA) eyes respectively.

Avg Δ (µm), abs value	Overall retina thickness, control	Overall retina thickness, DA	Inner retina thickness, control	Inner retina thickness, DA	Outer retina, thickness control	Outer retina thickness, DA
Average	2.57	2.61	2.40	2.16	1.56	1.79
St dev	1.58	1.33	1.35	1.067	0.94	0.75
P-value	0.89		0.35		0.20

Band intensities for the ellipsoid zone (EZ) band were also assessed and no significant changes were noticed (p = 0.46). The interdigitation zone (IZ) band length was measured as well and served as a proxy for determining the strength of the hyporeflective band between the inner segment (IS) and retinal pigment epithelium (RPE) bands. However, no IZ band changes were noted (p = 0.32).

## Discussion

Prolonged dark adaptation over a period of hours did not induce any significant thickness, volume, or intensity changes in the overall, inner, or outer retina in our healthy subjects; however, this was not the case in previous studies. Previous human OCT studies have shown that short periods of dark adaptation (the longest being 30 min) can lead to outer retinal thinning, thinning or absence of the hyporeflective band seen in light between the outer segment (OS) and RPE bands (which can also be interpreted as IZ band blurring), and inner segment ellipsoid (ISe) and EZ band intensity dimming in the dark; the same changes have been shown in mice for short dark adaptation periods (the longest being 2 h) and overnight dark adaptation^8,9,11,12,22^.

The lack of these changes could be due to several factors including homeostatic processes. Given the nature of homeostasis, it is possible that the photoreceptor dark current that causes the increased adenosine triphosphate (ATP) consumption in dark adaptation is not maintained over prolonged periods of time, as the eye has already acclimated to the dark. If the eye has already fully adjusted to its new light settings, there is no continued need to adjust, and retinal ATP consumption can return to its baseline levels. Furthermore, dark adaptation is known to slow with age^23^. Given our study participants were almost exclusively young, healthy adults, dark adaptation is expected to be faster. It is therefore possible that the previously reported dark adaptation structural changes are transient and occurred long before our four-hour dark adaptation period was completed in our young and healthy subjects, and then returned to baseline. Kim et al. performed dark adaptation over a five-minute period, imaging mice throughout the entirety of the dark adaptation period. They noted that band intensity changes generally came in phases, depending on the duration of dark adaptation; ISe intensity significantly reduced after only 5 min of dark adaptation, but inner plexiform layer (IPL), outer plexiform layer (OPL) and RPE band dimming only occurred at a later phase of dark adaptation^8^. Collectively, their data suggested that retinal changes occur linearly, and that the retina can respond incredibly quickly during dark adaptation. Dark adaptation recovery time in humans, however, has not yet been established in OCT studies. The lack of structural changes in our results necessitates further study to establish the longest dark adaptation period with structural changes.

However, mouse studies like Li et al. 2016 and Li et al., 2018 threaten to refute the possibility of quick dark adaptation recovery time in the setting of homeostatic processes^9,10^; overnight dark adaptation in these studies yielded structural changes as well, with a longer dark adaptation period that was also on the scale of several hours. While prolonged dark adaptation may well cause such structural changes, these changes may be due to other confounding variables, namely, diurnal rhythm. During disc shedding, photoreceptors are renewed through transient loss (then subsequent replacement) of the outer segment, thus leading to times of temporary outer segment shortening; as disc shedding is linked to the circadian cycle, the most active period of shedding is the early morning, and the least is in the late evening^24–30^. Therefore, any associated retinal thinning or shortening seen after an overnight period may be attributable to other confounding variables rather than dark adaptation.

Another potential variable that may account for the discrepancy between our data and those of previous studies is the imaging software used during OCT collection. Since the Spectralis system uses the fundal image as a reference during eye tracking, our study’s OCT image follow-ups were therefore performed at the same exact retinal location to allow for exact qualitative and quantitative comparison^10^. The Spectralis system uses the fundus image as a reference during eye tracking, so "follow-up mode" is a Spectralis feature that allows one to image the same exact retinal location upon later imaging sessions to allow for exact qualitative and quantitative comparison. Given that layer thickness varies across different retinal cross sections, eye tracking is a necessary tool for precise comparison between conditions. Since this feature is not widely available across different imaging systems, lack of eye tracking may account for some of these differences in results. As discussed in Krebs et al., variations in retinal thickness measurements can also be attributed to the different imaging systems’ methodologies, and therefore image quality, layer detection, and segmentation^31^. Other considerable differences are caused by localization control, scan line density, algorithms and segmentation line positioning.

In summary, we were unable to detect any significant retinal structural changes on OCT following four hours of dark adaptation in healthy humans. Further exploration of dark adaptation and retinal homeostasis, including analysis of choroidal thickness measurements and modulation of blood flow, may lend further insight into the mechanisms behind dark adaptation and prove clinically useful. Further studies across different age groups and with various intervals of dark adaptation, ranging on a scale of minutes to a few hours, and different times throughout the day and night, also should be performed to detect potential structural photoresponses, and if these photoresponses can indeed serve as future clinical tools in diagnosing and treating retinal pathologies.

## Methods

### Dark adaptation and OCT imaging protocol

**Figure 3:**
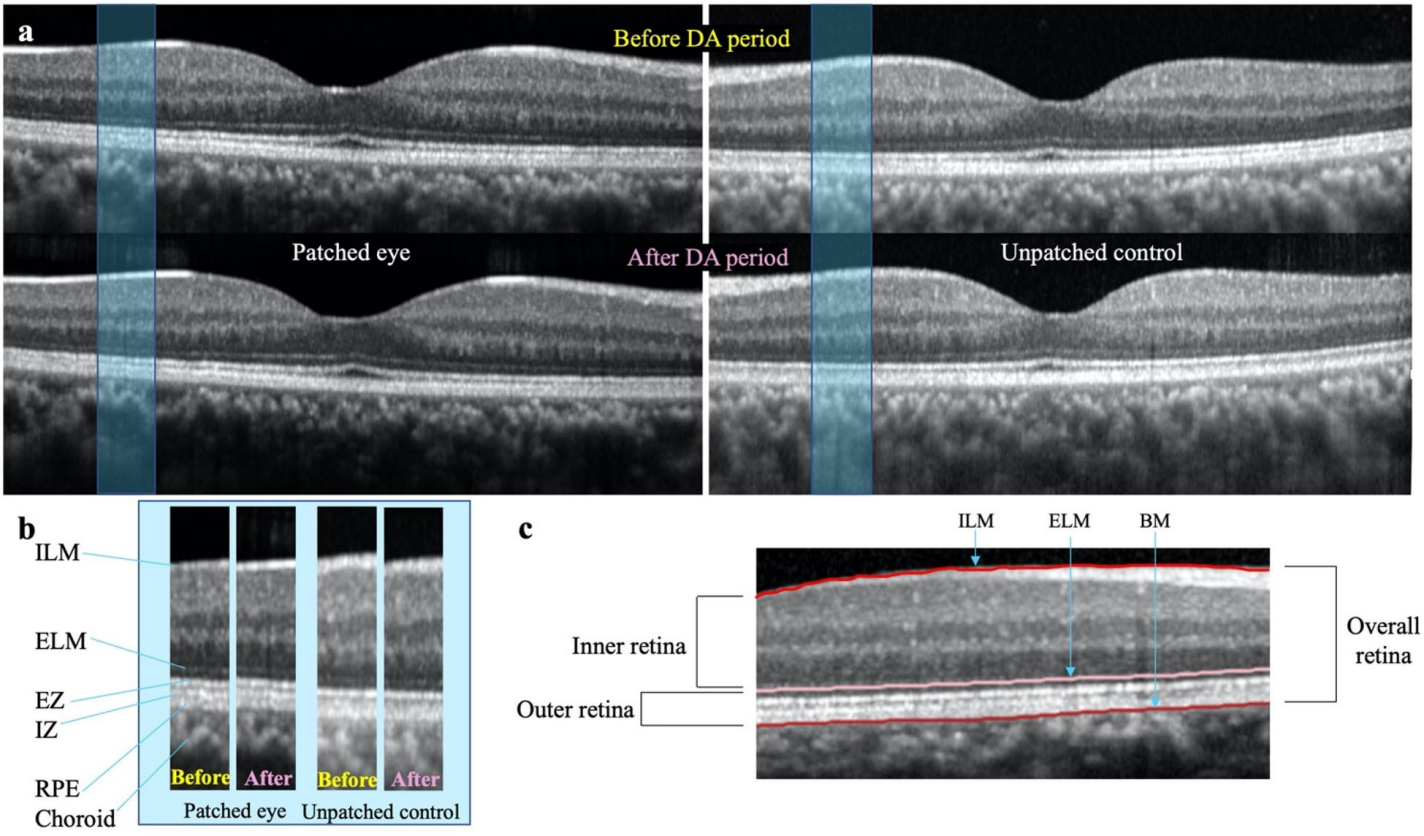
Example of Spectral Domain Optical Coherence Tomography (SD OCT) retinal images obtained (**A**) B-scan of one subject’s retina through the fovea in the dark adapted/patched eye (left) and unpatched control eye (right), before and after the dark adaptation (DA) period. (**B**) Side-by-side comparison of the OCT images for the same local retinal region (outlined by the blue rectangles in [**A**]) for patched and unpatched eyes, before and after the DA period. *ILM* internal limiting membrane, *ELM* external limiting membrane, *EZ* ellipsoid zone, *IZ* interdigitation zone, *RPE* retinal pigment epithelium (**C**) Example of the segmentation performed by the Heidelberg Spectralis software that was used to generate thickness maps for the outer retinal layer. *BM* basement membrane.

OCT images were taken from both eyes at baseline and four hours later. Between the two OCT imaging, one eye was dark adapted and the other eye was kept as control (Fig. 3A,B). The baseline OCT image of both eyes were taken in the dark between 12 p.m. and 1 p.m. to avoid peak disc shedding time as a possible confounder, similar to Lu et al.^22^. Subjects who wore contact lenses were instructed to remove their contact lenses for the duration of the experiment. Subjects who wore glasses were instructed to remove their glasses during OCT imaging and could elect to wear their glasses during the dark adaptation period over the eye patch if they so chose.

After this initial baseline OCT image was taken, we randomly selected one of each subject’s two eyes and covered that eye with an eye patch that was designed specifically for this study and blocked all potential light from getting through. The eye patch involved taping three layers to the area around the subject’s selected eye: first, a disposable cotton eye pad; second, a flexible sheet of black plastic impenetrable to light; third, a final layer of gauze. After the addition of each eyepatch layer, the subject was asked to shut the other unpatched eye and confirm that no light was visible through or around the eye covering.

The subjects were instructed to keep the eye patch on for four hours in between the two imaging sessions. During this time, the subjects were instructed to stay in a relatively well-lit setting and were instructed to not sleep or spend the patching period in a dark place. These instructions were given to ensure the unpatched eye would be exposed to light for the full duration of the experiment as opposed to the patched eye, which would remain in the dark throughout the experiment. Of the 40 participants, 20 were observed throughout the entirety of the four-hour dark adaptation period and were confirmed to have followed instructions. The remaining 20 gave verbal affirmation they followed instructions. After the dark adaptation period was over, the subjects returned for a second OCT imaging session where both eyes imaged again. The room was kept dark during both imaging sessions to ensure higher quality OCT images. The room was also kept dark during imaging to ensure the patched eye remained dark adapted during post-patching imaging and was not exposed to light until after the patch was removed. The dark adapted eye was imaged immediately after removal of the patch. No follow-up was needed with the subjects after their OCT images were collected.

### OCT settings

The Spectralis SD OCT instrument (Heidelberg Engineering, Heidelberg, Germany) was used to obtain 20° × 20° OCT cube scans (49 sections, 1024 A-scans in each B-scan, Automated Real Time 20 frames) centered on the fovea of each eye. Images of each eye, both before and after patching, were taken. During the study period, all scans were performed using the follow-up mode after setting the scans before the dark adaptation period as reference. Images of two subjects were eliminated from the study due to lack of using follow-up mode (Table 1).

### Analysis

Segmentation of retinal layer boundaries on each OCT retinal B-scan and thickness maps of the desired retinal cell layers were performed and generated by Heidelberg software (Fig. 3C). Internal limiting membrane (ILM), external limiting membrane (ELM) and basement membrane (BM refers to the basement membrane of the choriocapillaries/Bruch’s membrane) were used for segmentation. The Heidelberg Spectralis segmentation software reliably detects these structures and provides accurate segmentation. Given that previous studies have shown significant thickness changes in the outer retinal layer, for the thickness maps, the retinal cell layers chosen for analysis were the outer retinal layer (between BM and ELM), inner retinal layer (between ELM and ILM), and overall retina (between BM and ILM).

For each analyzed retinal layer, the thicknesses across the 49 scans were averaged across nine regions of interest, including the foveal and the inner and outer perifoveal (superior, nasal, inferior, and temporal) regions (Fig. 1A, Supplementary Figs. 1–4A).

Averaged thicknesses across the foveal and inner and outer perifoveal regions were compared before and after dark adaptation for each subject. The absolute values of the before-and-after differences were compared between dark adapted/patched versus unpatched control eyes by paired t testing, two-tailed. Statistical significance was defined as p < 0.05.

For band intensity evaluation, all B-scans for the dark adapted/patched and unpatched control eyes, before and after the dark adaptation period, were masked and quantitatively and assessed for their EZ and IZ band intensities.

For assessing EZ band intensity changes, we used EZ:ELM ratio to eliminate potential intensity changes introduced to each scan because of factors such as media clarity, head tilt etc. Invitrogen GelQuant Express software was used to evaluate EZ band intensity changes over the central 2600 µm region on the central line scan. This region was split into 11 boxes, and due to irregular contour of bands in the fovea, the central-most box was excluded from analysis. The EZ and ELM band intensities were measured and the EZ:ELM ratios were calculated and averaged across the remaining 10 boxes. The EZ:ELM ratios, after vs before the dark adaptation period, were compared between light and dark adaptation conditions using paired t-testing, two-tailed.

In addition to EZ band intensity, we evaluated OCT images for any clinically significant changes. In a pilot observation we noticed some changes in the length of visibility of IZ band before and after dark adaptation. Thereafter, the IZ band lengths, before and after the dark adaptation period, were compared using paired t-testing, two-tailed.

### Ethics declaration

This methodology has been approved by the USC Biomedical Institutional Review Board ethics committee (HS-21-00842) in accordance with current guidelines and regulations. Informed consent was obtained from all participants.

## Supplementary Information

Supplementary Figure 1.

Supplementary Figure 2.

Supplementary Figure 3.

Supplementary Figure 4.

Supplementary Table 5.

Supplementary Table 6.

## Data Availability

The (de-identified) datasets generated during and/or analyzed during the current study are available from the corresponding author on reasonable request.
