# Supplementary material for: Optical coherence tomography in healthy human subjects in the setting of prolonged dark adaptation
Source: Sci Rep. 2023 Mar 6;13:3725. doi: 10.1038/s41598-023-30747-0 (PMC9988879; doi:10.1038/s41598-023-30747-0)
Supplement: Supplementary file 1 — Supplementary Figure 1. [file 41598_2023_30747_MOESM1_ESM.docx]

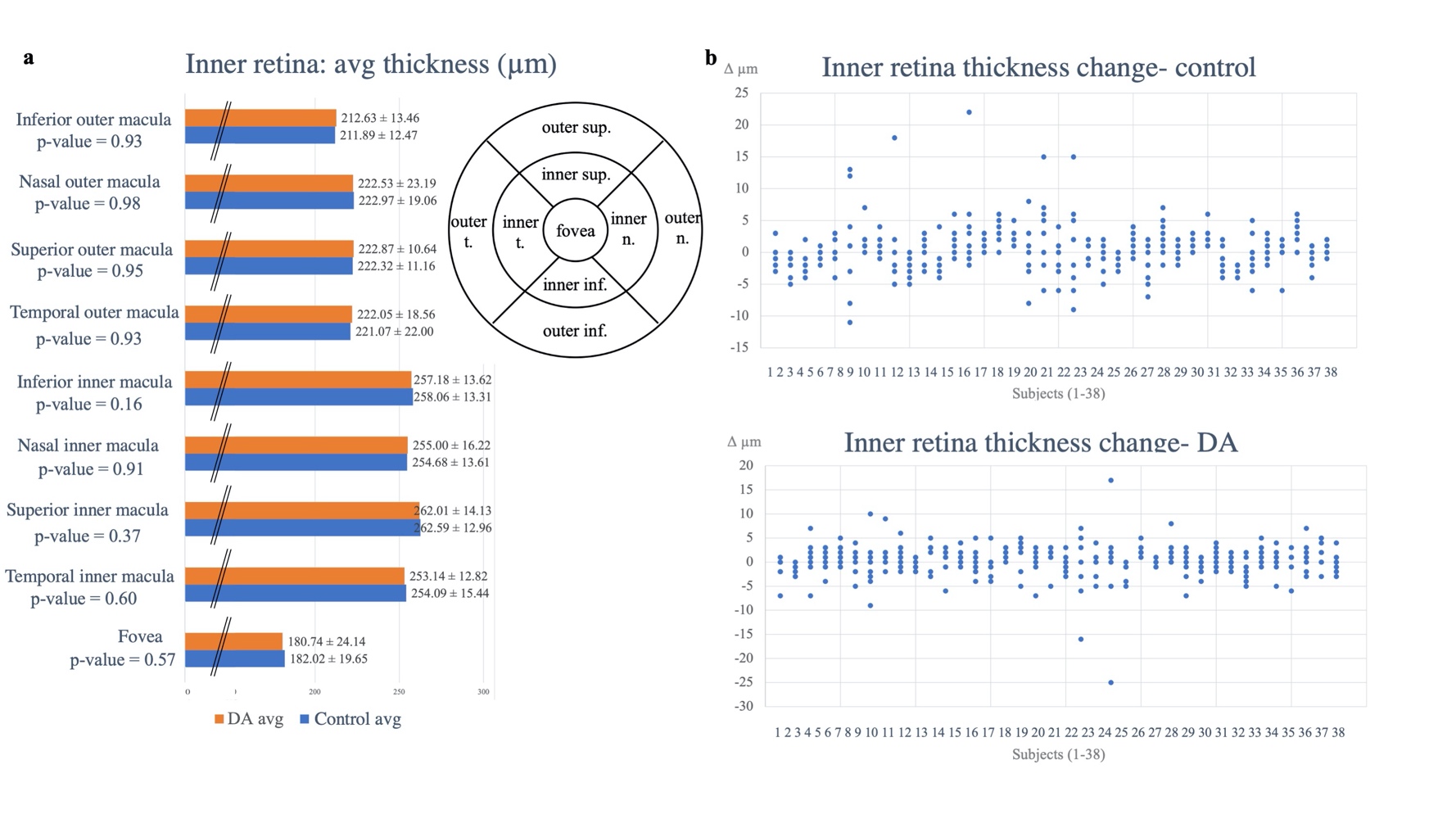


Supplemental Figure 1: Inner retinal thickness measurements before and after dark adaptation (DA) at baseline and four hours later in the dark adapted and control eyes. (A) Bar graph comparing dark adapted (orange) and control (blue) average thickness measurements for the 9 regions of interest (foveal, and inner and outer perifoveal regions), with a labelled thickness map example template included. (B) Average differences for the inner retinal layer for control and dark adaptation conditions respectively.
