# Supplementary material for: Optical coherence tomography in healthy human subjects in the setting of prolonged dark adaptation
Source: Sci Rep. 2023 Mar 6;13:3725. doi: 10.1038/s41598-023-30747-0 (PMC9988879; doi:10.1038/s41598-023-30747-0)
Supplement: Supplementary file 3 — Supplementary Figure 3. [file 41598_2023_30747_MOESM3_ESM.docx]

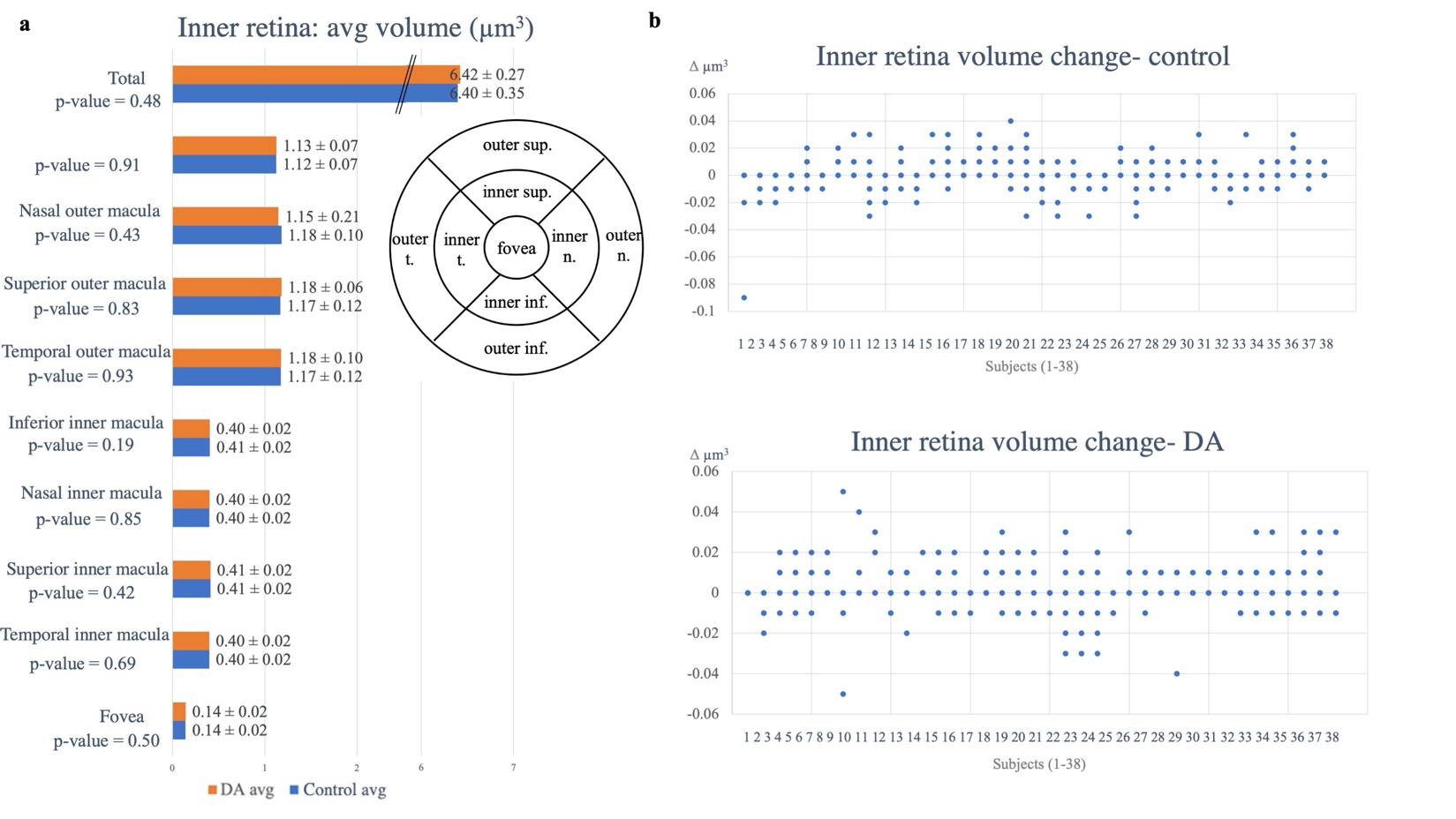


Supplemental Figure 3: Inner retinal volume measurements before and after dark adaptation (DA) at baseline and four hours later in the dark adapted and control eyes. (A) Bar graph comparing dark adapted (orange) and control (blue) average thickness measurements for the 9 regions of interest (foveal, and inner and outer perifoveal regions) and total volume measurement average, with a labelled thickness map example template included. (B) Average differences for the inner retinal layer for control and dark adaptation conditions respectively. One outlier in the DA condition was omitted to allow for the detection of smaller differences among the other subjects.
