# Supplementary material for: Optical coherence tomography in healthy human subjects in the setting of prolonged dark adaptation
Source: Sci Rep. 2023 Mar 6;13:3725. doi: 10.1038/s41598-023-30747-0 (PMC9988879; doi:10.1038/s41598-023-30747-0)
Supplement: Supplementary file 5 — Supplementary Table 5. [file 41598_2023_30747_MOESM5_ESM.docx]

| **Region of interest** | **Avg volume (µm^3^)** | **Overall retina** | **Inner retina** | **Outer retina** |
| --- | --- | --- | --- | --- |
| **Fovea** | DA  Control  p-value | 0.22 ± 0.02  0.22 ± 0.02  p = 0.99 | 0.14 ± 0.02  0.14 ± 0.02  p = 0.50 | 0.07 ± 0.004  0.07 ±0.003  p = 0.18 |
| **Temporal inner macula** | DA  Control  p-value | 0.53 ± 0.02  0.53 ± 0.03  p = 0.57 | 0.40 ± 0.02  0.40 ± 0.02  p = 0.69 | 0.13 ± 0.01  0.13 ± 0.01  p = 0.87 |
| **Superior inner macula** | DA  Control  p-value | 0.54 ± 0.02  0.54 ± 0.02  p = 0.52 | 0.41 ± 0.02  0.41 ± 0.02  p = 0.42 | 0.13 ± 0.01  0.13 ± 0.01  p = 0.37 |
| **Nasal inner macula** | DA  Control  p-value | 0.53 ± 0.05  0.54 ± 0.02  p = 0.55 | 0.40 ± 0.02  0.40 ± 0.02  p = 0.85 | 0.13 ± 0.01  0.13 ± 0.01  p = 0.82 |
| **Inferior inner macula** | DA  control  p-value | 0.53 ± 0.02  0.54 ± 0.02  p = 0.30 | 0.40 ± 0.02  0.41 ± 0.02  p = 0.19 | 0.13 ± 0.01  0.13 ± 0.01  p = 0.52 |
| **Temporal outer macula** | DA  control  p-value | 1.61 ± 0.10  1.59 ± 0.13  p = 0.80 | 1.18 ± 0.10  1.17 ± 0.12  p = 0.93 | 0.43 ± 0.03  0.43 ± 0.02  p = 0.11 |
| **Superior outer macula** | DA  control  p-value | 1.61 ± 0.06  1.61 ± 0.06  p = 0.32 | 1.18 ± 0.06  1.17 ± 0.12  p = 0.83 | 0.43 ± 0.02  0.43 ± 0.02  p = 0.054 |
| **Nasal outer macula** | DA  control  p-value | 1.60 ± 0.13  1.61 ± 0.10  p = 0.97 | 1.15 ± 0.21  1.18 ± 0.10  p = 0.43 | 0.42 ± 0.02  0.42 ± 0.02  p = 0.63 |
| **Inferior outer macula** | DA  Control  p-value | 1.54 ± 0.08  1.54 ± 0.07  p = 0.80 | 1.13 ± 0.07  1.12 ± 0.07  p = 0.91 | 0.42 ± 0.02  0.42 ± 0.02  p = 0.17 |
| **total** | DA  Control  p-value | 8.71 ± 0.33  8.68 ± 0.41  p = 0.33 | 6.42 ± 0.27  6.40 ± 0.35  p = 0.48 | 2.30 ± 0.09  2.28 ± 0.10  p = 0.078 |

Supplemental Table 1: Average volume measurements and p-values for all 9 regions of interest (foveal and inner and outer perifoveal regions) between control and dark adaptation (DA) conditions.
