# Supplementary material for: Optical coherence tomography in healthy human subjects in the setting of prolonged dark adaptation
Source: Sci Rep. 2023 Mar 6;13:3725. doi: 10.1038/s41598-023-30747-0 (PMC9988879; doi:10.1038/s41598-023-30747-0)
Supplement: Supplementary file 6 — Supplementary Table 6. [file 41598_2023_30747_MOESM6_ESM.docx]

| **Avg Δ (µm^3^), abs value** | **Overall retina volume, control** | **Overall retina volume, DA** | **Inner retina volume, control** | **Inner retina volume, DA** | **Outer retina, volume control** | **Outer retina volume, DA** |
| --- | --- | --- | --- | --- | --- | --- |
| average | 0.0077 | 0.0087 | 0.0068 | 0.013 | 0.13 | 0.0058 |
| St dev | 0.0057 | 00.0051 | 0.0032 | 0.025 | 0.077 | 0.0032 |
| P-value | 0.39 |  | 0.14 |  | 0.33 |  |

Supplemental Table 2: Absolute values of average change in volumes, standard deviations, and p-values for overall, inner, and outer retinal layers between control and dark adaptation (DA) conditions.
